# Transcriptomic Analysis of *Fusarium oxysporum* Stress-Induced Pathosystem and Screening of *Fom-2* Interaction Factors in Contrasted Melon Plants

**DOI:** 10.3389/fpls.2022.961586

**Published:** 2022-07-22

**Authors:** Tiantian Yang, Jiajun Liu, Xiaomei Li, Sikandar Amanullah, Xueyan Lu, Mingchong Zhang, Yanhang Zhang, Feishi Luan, Hongyu Liu, Xuezheng Wang

**Affiliations:** ^1^College of Horticulture and Landscape Architecture, Northeast Agricultural University, Harbin, China; ^2^Key Laboratory of Biology and Genetic Improvement of Horticulture Crops (Northeast Region), Ministry of Agriculture and Rural Affairs, Harbin, China; ^3^College of Life Sciences, Northeast Agricultural University, Harbin, China

**Keywords:** melon, transcriptome, *Fusarium oxysporum* (f. sp. *melonis* race 1), *Fom-2*, yeast two-hybrid

## Abstract

Fusarium wilt is one of the most destructive and less controllable diseases in melon, which is usually caused by *fusarium oxysporum*. In this study, transcriptome sequencing and Yeast Two-Hybrid (Y2H) methods were used for quantification of differentially expressed genes (DEGs) involved in *fusarium oxysporum* (f. sp. *melonis* race 1) stress-induced mechanisms in contrasted melon varieties (M4-45 “susceptible” and MR-1 “resistant”). The interaction factors of *Fom-2* resistance genes were also explored in response to the plant-pathogen infection mechanism. Transcriptomic analysis exhibited total 1,904 new genes; however, candidate DEGs analysis revealed a total of 144 specific genes (50 upregulated and 94 downregulated) for M4-45 variety and 104 specific genes (71 upregulated and 33 downregulated) for MR-1 variety, respectively. The analysis of Kyoto Encyclopedia of Genes and Genomes (KEGG) pathway depicted some candidate DEGs, including Phenylalanine metabolism, phenylpropane biosynthesis, plants-pathogen interaction, and signal transduction of plant hormones, which were mainly involved in disease resistance metabolic pathways. The weighted gene co-expression network analysis (WGCNA) analysis revealed a strong correlation module and exhibited the disease resistance-related genes encoding course proteins, transcription factors, protein kinase, benzene propane biosynthesis path, plants-pathogen interaction pathway, and glutathione S-transferase. Meanwhile, the resistance-related specific genes expression was relatively abundant in MR-1 compared to the M4-45, and cell wall-associated receptor kinases (*MELO3C008452* and *MELO3C008453*), heat shock protein (Cucumis_melo_newGene_172), defensin-like protein (Cucumis_melo_newGene_5490), and disease resistance response protein (*MELO3C016325*), activator response protein (*MELO3C021623*), leucine-rich repeat receptor protein kinase (*MELO3C024412*), lactyl glutathione ligase (Cucumis_melo_newGene_36), and unknown protein (*MELO3C007588*) were persisted by exhibiting the upregulated expressions. At the transcription level, the interaction factors between the candidate genes in response to the *fusarium oxysporum* induced stress, and Y2H screening signified the main contribution of MYB transcription factors (*MELO3C009678* and *MELO3C014597*), BZIP (*MELO3C011839* and *MELO3C019349*), unknown proteins, and key enzymes in the ubiquitination process (*4XM334FK014*). The candidate genes were further verified in exogenously treated melon plants with *f. oxysporum* (*Fom-2*, Race 1), Abscisic acid (ABA), Methyl Jasmonite (MeJA), and Salicylic acid (SA), using the fluorescence quantitative polymerase chain reaction (qRT-PCR) analysis. The overall expression results indicated that the SA signal pathway is involved in effective regulation of the *Fom-2* gene activity.

## Introduction

Melon (*Cucumis melo* L.) is an important Cucurbit crop, which is widely cultivated around the world. China alone produces greater than one-third of worldwide production for cucurbit crops, contributing 49.10% of melon production (Grumet et al., 2021). The planting area, average yield, and other commercial benefits of melon are increasing day by day, but the yield-limiting pathogen “*fusarium oxysporum*” is also becoming very serious and greatly hindering the sustainable development of the melon industry (Branham et al., 2021). However, there are numerous abiotic (radiation, salinity, floods, drought, extremes in temperature, heavy metals) and biotic (pathogens attacks, e.g., fungi, bacteria, oomycetes, nematodes, and herbivores) stresses that influence the plant growth and reduce the crop productivity (Fahad et al., 2013; Fahad et al., 2014a,b; Atif et al., 2021).

Fusarium wilt is a well-known disease at a global scale, which is mainly activated by *fusarium oxysporum* infection. In 1933, this disease was initially discovered in the United States, mainly caused by Race 0, Race l, Race 2, and Race 1.2 (Risser et al., 1976) and steadily spread in many countries (Kurt et al., 2002). These main races were widely distributed in the Europe and Middle East territories and also named as “common physiological races” (Moretti et al., 2002). Later, the disease symptoms caused by Race 1.2 were further subdivided into parts “l.2w (causes wilting) and 1.2y (causes yellowing)” (Namiki et al., 1998b; Hela et al., 2013). In Japan, Race 0 and Race 1 were known as major comparative races (Namiki et al., 1998a), and melon variety (MR-1) was reported as a main concern in the disease host system (Gordon and Martyn, 1997). There are three known resistance genes in response to the *fusarium oxysporum* infection in melon. The *Fom-1* gene is resistant to Race 0 and Race 2 but susceptible to Race 1 and Race 1.2 (Risser et al., 1976; Oumouloud et al., 2010), and *Fom-3* is resistant to Race 0, Race 1, and Race 2, but also susceptible in response to Race 1.2 (Zink and Gubler, 1985); however, two other resistance-related genes “*Mc* and *MC-2*” have been discovered in response to the melon resistance mechanism (Herman and Perl-Treves, 2007). A total of 11 *Fom-2* gene sequences have been recorded in the GenBank database (Joobeur et al., 2004; Garcia-Mas et al., 2012), and *Fom-1* and *Fom-2* genes have been successfully cloned (Schmidt et al., 2016), but still need in-depth clarification for identifying the resistance and susceptible mechanisms.

The melon resistant varieties were inoculated with *fusarium oxysporum*, and *CmCAD2, CmCAD3, CmCAD5* genes expression was found asup-regulated inroot and leaf tissues, suggesting the possible response of lignin defense pathway (Liu H. J. et al., 2016; Liu et al., 2020). Further, the molecular mechanism of resistant variety “NAD” and susceptible variety “Charentais-T (CHT)” was identified by infecting with race1.2 and NAD variety rapidly activated its defense response through early recognition of pathogen-related defense mechanisms. This response was seemed to be associated with Ca^2+^ that triggered the signaling pathways, cell-wall recombination, and well regulation of hormone interactions (jasmonic acid and ehylene, auxin and abscisic acid (ABA)] (Sebastiani et al., 2017). Numerous studies exposed that relative gene expression of ethylene synthesis significantly increases the *fusarium oxysporum* response (Lanubile et al., 2015). The resistance mechanism of seven major genes showed significant results of melon-*fusarium oxysporum*-induced stress in herbicide, including zinc finger DNA-binding protein, ADP-glucose-pyrophosphorylase, terpenoid synthase, glycolate oxidase, and 1-deoxy-D-xylose-5-phosphate reductisomerase (Lotan-Pompan et al., 2007). The late expression of 300 DEGs in the host interaction caused the infection of two physiological races was studied, and the host genome exhibited 50 fragments that were homologous to *f. oxysporum*; however, the expression level was noticed as increased, and no expression was detected in the pathogen culture (Szafranska et al., 2008).

Next-generation sequencing (NGS) has provided new methods of transcriptome analysis, termed as RNA sequencing (RNA-Seq). This sequencing approach provides a precise measurement for transcript levels to reveal the responsive mechanisms toward specific stimuli (Zhu et al., 2017). In the last decade, RNA-Seq has been widely used for in-depth molecular understanding of various adaptive evolution, host immune system, and stress responses in many living organisms (Qian et al., 2014); e.g., rainbow trout (*Salmo gairdneri*) (Salem et al., 2016), fish species (Long et al., 2012), sea bream *Sparu aurata* (Calduch-Giner et al., 2014), sea bass (*Lateolabrax japonicas*) (Magnanou et al., 2014), and large yellow croaker (*Larimichthy crocea*) (Qian and Xue, 2016). RNA-seq has been similarly used for comparative transcriptomic analysis of morphology and quality-related significant traits of cucurbit fruit crops, e.g., fruit ripening-related climacteric behavior and flesh hardness of watermelon (*Citrullus lanatus* L.) (Zhu et al., 2017; Sun et al., 2020), and the powdery mildew resistance mechanism in melon (*Cucumis melo* L.) (Zhao et al., 2022), plants-pathogen interactions, and investigation of disease resistance or the susceptibility mechanism (Szafranska et al., 2008; Balcke et al., 2017; Yang et al., 2021). In melon, the *de novo* sequenced melon genome (Garcia-Mas et al., 2012) has efficiently accompanied in boosting the well usage of transcriptome sequencing (RNA-seq), which identified an in-depth regulatory mechanism of underlying genes affecting the various traits.

Differentially expressed genes (DEGs) involved in various biological processes can be categorized by the RNA-seq method. The elucidation of these changes at the transcriptome level would facilitate the in-depth understanding of key biological and physiological mechanisms (Huang et al., 2018). These studies also identified the candidate regulatory pathways involved in response to heat stress, including metabolism, protein folding and degradation, and immune response, indicating that these biological pathways are critical to regulating the significant traits. Furthermore, weighted gene co-expression network analysis (WGCNA) is a method frequently used to explore the complex relationships between genes and phenotypes. It has been widely used to analyze the high-throughput sequencing data (Ruan et al., 2010), which aided in the identification of key genes involved in development of a peculiar trait (Perumal et al., 2014).

In addition, the Y2H method has been used for identification of unknown protein interactions in different crop plants, e.g., rice and wheat (Niu et al., 2016; Wang et al., 2016; Wang X. et al., 2018). This method is an effectual approach to identify the protein in genetic system of living cells and to study the interaction between proteins (Fields and Song, 1989; Shazadee et al., 2022). This Y2H technique cannot only measure the interaction between known proteins but also reveals the unknown proteins interaction with known proteins by screening the suitable yeast libraries. In 2001, researchers completed the first plant-protein interaction network map the “*Arabidopsis thaliana* interaction map,” which provided a great significance to understand the relationship between plant proteins (Dreze et al., 2011). In watermelon, decoy proteins were constructed using fusarium-induced pathogenic factor “*FonSIX6*,” and 14 interacting target proteins were screened from the cDNA expression library using the Y2H method (Niu et al., 2016). It was found that the target site of *FonSIX6* is mainly helpful to destroy the energy supply system of the host to act on photosynthesis, and the host responded toward regulating the expression of disease-resistant genes. It was also speculated that screened proteins were mainly contributed in different energy pathways of metabolism, photosynthesis, and regulation of host-related gene expressions.

In melon (*Cucumis melo* L.), *fusarium oxysporum* causes serious damage to crops, and numerous breeding works have been done for developing the resistant melon varieties based on identifying and incorporating the known resistance genes, but the resistance mechanism is still not clear. The discovery of new key genes regulating the signaling pathway will put some new clues for in-depth genetic understanding of melon resistance toward *fusarium oxysporum*. There is a dire need to know how the resistance genes are involved in melon-*fusarium oxysporum* resistance, what are the expression characteristics and the molecular mechanism of resistance, and which signal pathways are involved in response to the melon-*fusarium oxysporum* stress-induced mechanism in melon. Hence, in the current study, transcriptome sequencing was carried out for contrasted melon (resistance and susceptible varieties), candidate DEGs were screened out, and the molecular mechanism of melon’s response toward *fusarium oxysporum*-induced stress was elucidated. In addition, the Y2H method was applied for screening the interacting protein with resistance gene “*Fom-2*” from the melon*-fusarium oxysporum-*induced cDNA library.

## Materials and Methods

### Experiment Materials and Inoculation Stress

Two contrasted melon varieties (“M4-45” susceptible and “MR-1” resistant) with different disease responding mechanisms toward *fusarium oxysporum* (f. sp. *melonis* (Race 1) were chosen for experimental validation. These materials were obtained from Horticulture College, Northeast Agricultural University, Harbin, China. The race 1 hyphae were inoculated from a potato-dextrose agar (PDA) plate using a potato lactose medium, incubated at 25°C for 7 days before being cut into0.5-cm^2^ cubes, inoculated in a liquid medium [1.1-g KH_2_PO_4_, 1.6-g KCl, 5.9-g Ca(NO_3_)_2_, 0.4-g MgSO_4_⋅7H_2_O, 0.2-mg ZnSO_4_, 0.2-mg MnSO_4_, 0.2-mg FeCl_3_, 20-g Glucose; distilled water was added up to 1 L], and stirred in a magnetic stirrer (120 rpm) at 27°C for 3 days, respectively (Wu et al., 2013).

The primary seedlings were grown up to a two-cotyledon stage, and then plantlets were inoculated by dipping in the prepared conidial suspension (1 × 10^6^ spore/ml) for a total of 15 min, and water-inoculated seedlings were used as mock controls (CK). Later, all the inoculated seedlings were shifted into 10 cm × 10 cm plastic-pots filled with sterilized soil, and optimum temperature (25 ± 2°C), with 80–90% relative humidity was maintained in the greenhouse. A completely randomized design (CRD) was adopted by following 3 replicates in each treatment, and each replication contained 15 plants, respectively. The emerged root tissues were taken at three different time intervals of fungal stress at 0 h (CK), 12 and 72 h; roots were rinsed with distilled water, and dried with moist filter paper, and then preserved at suitable temperature.

### Total RNA Isolation and Illumina Sequencing

The treated samples of both melon materials “M4-45 and MR-1” were chosen at contrasted time intervals of inoculated stress treatment and named into two groups “ACK, A12 h, A72 h; and BCK, B12 h, B72 h”, respectively. Total RNA was isolated from 18 samples (3 replicates of each treatment) using the Trizol reagent (Invitrogen) (Yang et al., 2022). The RNA quantification was done using the NanoDrop 2000 (Thermo Fisher Scientific) and evaluated through the RNA Assay Kit (Nano 6000) in the Agilent Bioanalyzer 2100 System (Agilent Technologies, United States). Then, a sequencing library was constructed using the NEBNext Ultra-TM-RNA Library Preparation Kit for sequencing library construction. After the complete passing of quality and inspection, sequencing was done using an Illumina sequencing platform by Beijing Baimaike Biotechnology Co., LTD. The raw RNA-seq data were uploaded to the online Sequence Read Archive (SRA) database (PRJNA842515, including 16 biosamples in the bio project) of the National Center for Biotechnology Information (NCBI).

### Mapping of RNA Sequencing Reads and Differentially Expressed Genes Calling

To get the clean reads, raw fast-Q data files were cross-checked (quality ≤ 20); the unsuitable raw reads were cropped with Trimmomatic-0.30 (Bolger et al., 2014). The filtered reads were mapped over *de novo* assembled melon genome (DHL92, v3.5.1) (Garcia-Mas et al., 2012) using the HISAT2 software, and sequences of newly identified genes were compared using BLAST (Altschul et al., 1997). The KEGG ortholog results of the new genes were obtained by KOBAS2.0 (Xie et al., 2011), and final genes were predicted for amino acid sequences analysis, and compared with the database as follows: NCBI nr, Pfam, KOG, Swiss-Prot, KEGG, COG, GO, and melon genome.

The obtained single-end raw sequencing reads were checked, low quality reads mapped to the melon transcriptome for estimation of transcript abundance per individual by using the Bowtie2 (Langmead and Salzberg, 2012). To count the variability of total initial Illumina sequencing results among the samples, mapped read counts were subjected to the normalization process, and then DEGs were called using the DESeq2 Bioconductor package (v1.2.8) (Love et al., 2014). To detect the primary DEGs in melon (control vs. inoculated plants of both melon genotypes), the DESeq cut off (0.5 RPKM) was set for considering the expressed DEGs, while the DESeq parameters for dispersion deliberation were set with method ‘‘pooled’’ and sharing Mode ‘‘fifitOnly.’’ The threshold score of false discovery rate (FDR, 0.05) for DEG calling was set to 0.05. All *Fom-2*-related DEGs with a log2 fold change (log2-fc) ‘‘different from NA’’ were considered as expressed. DESeq-normalized melon samples were transformed with the function VST ‘‘DESEq package,’’ and Venn diagrams were generated by the Venny tool^1^ using DUR transcripts, resulting from DESeq2 by comparing each resistant and susceptible variety.

### Weighted Gene Co-expression Network Analysis

The co-expression networks were constructed using the weighted gene co-expression network analysis (WGCNA, v1.47) package in the R language programming tool (v4.2.0) (Kogelman et al., 2014). After filtering the genes, gene expression values were imported into WGCNA, and co-expression modules were constructed. The expression correlation coefficients of the remaining genes were then calculated to search a suitable soft threshold for building gene networks using a scale-free topology model (Wang N. et al., 2018). Then, module eigenes were used to calculate the correlation coefficients, and biologically significant modules were identified. Intramodular connectivity (function soft connectivity) of each gene was calculated, and the top 1 or 5% of genes with the highest connectivity tended to be candidate genes using Cytoscape_3.3.0. For identification of the genes in each module, GO enrichment pathway analyses were conducted to analyze the biological functions of the modules, and *q*-value < 0.05 was used as the threshold after correction.

### Construction of *Fom-2* Decoy Protein, Self-Activation, and Toxicity Identification

The plasmid vector “PMD18-T-FOM-2” of *Fom-2* gene was also provided by College of Horticulture, Northeast Agricultural University, Harbin. In brief, the plasmids “pGBKT7 and PMD18-T-FOM-2” were double digested through *Eco*RI and *Bam*HI endonucleases, and the *Fom-2* gene and the pGBKT7 vector were obtained. These two vectors were successfully ligated with T4-DNA ligase and then transferred to *E. coli* for culturing. The plasmid was extracted from bacterial liquid containing a target fragment, identified by enzyme digestion, then sequenced by Shanghai Sangong Bioengineering Co., LTD., and, finally, identified plasmid was named as pGBKT7-*Fom*-2.

The bait vector (pGBKT7-*Fom*-2), blank control (pGBKT7), positive control plasmid (PGBKT7-53), and negative control plasmid (PGBKT7-LAM) were transformed into yeast-competent cells by the PEG/Li Ac method. Then, Y2HGold (PGBKT7-*Fom*-2) was coated on SD/-TRP, SD/-TRP/X-A-Gal, SD/-TRP/AbA, SD/-TRP/-ADE, and SD/-TRP/-HIS mediums. The male-positive control Y2HGold (PGBKT7-53) × Y187 (pGADt7-T), and male-negative-control Y2HGold (PGBKT7-lam) × Y187 (pGADt7-T) were mated in the small groups, respectively. They were spread on SD/-LEu/-TRP and SD/-LEu/-TrP/X-A-Gal/AbA nutrient-deficiency mediums as a control test in subsequent mating, cultured at 30°C for 3–5 days, and allowed to detect the self-activation growth.

### Screening of Complementary DNA Library and Yeast Two-Hybrid Assay

The interaction between bait and library plasmids was identified using the three-frame Y2H cDNA library of melon *f. oxysporum*, constructed in our own laboratory (Luan et al., 2019). The Y2H assay was based on the Matchmaker Gold Yeast-hybrid system of Y2HGold (pGBKT7-*Fom*-2). The blue colonies grown on a DDO/X/A nutrient deficiency plate were chosen and inoculated on a QDO/X/A nutrient-deficiency plate for more stringent screening. These blue colonies were then cultured. Yeast plasmid was extracted, and bait protein interaction was checked with the library protein. The extraction method was based on Easy Yeast Plasmid Isolation Kit, and pGADT7-related primers (Forward-GGAGTACCCATACGACGTACC and Reverse-TATCTACGATTCATCTGCAGC) were used for detection of yeast colony using PCR amplification. The pGBKT7 + AD-X, positive retest, pGBKT7-FOM-2 + AD-X, pGBKT7-53 + pGADT7-T were used as positive (+) control for interaction and AD-X self-activation detection, and pGBKT7-Lam + pGADT7-T were applied as a negative (−) control for interaction.

After screening the interacting proteins, further verification of reverse hybridization, pull down, and immuno-precipitation was needed. So, we used the reverse hybridization method based on the sequencing results, and target fragments of AD and BD decoy plasmids were transformed into two yeast strains “Y187 and Y2HGold,” respectively. The interaction between selected targeted protein and BD decoy protein was confirmed once more by hybridizing the Y187 yeast with AD plasmid, and Y2HGold yeast with BD decoy plasmid.

### Complementary DNA and Quantitative Polymerase Chain Reaction for Gene Validation

First-strand cDNA was synthesized from 100 ng of total RNA using the TRUEscript 1st Strand cDNA Synthesis Kit (Aidlab, Beijing, China). Total 10 DEGs, which were characterized by their expression profiles in response to the compatible and incompatible combinations with *f. oxysporum* (f. sp. *melonis* Race1) infection, were selected to verify the quality of transcriptome sequencing results using the qRT-PCR method. The molecular primers were designed using the Primer Premier v6.0 software (Supplementary Table 1), primers specificity was checked by blasting their sequences using the online NCBI database, and actin gene (*MELO3C023264*) was used as an internal control gene.

The candidate gene primer sequences (sense + anti-sense primers) were designed (Supplementary Table 2), and employed for qRT-PCR analysis. All reactions were carried out on an iQ5 Gradient Real Time PCR system (Bio-Rad, CA, United States) (Sebastiani et al., 2017; Yang et al., 2022), using 10-μl reaction mixture, comprising of 30-ng cDNA, 0.2-μl per primer, and 5 μl of the SYBR Green Realtime PCR Master Mix (TOYOBO, Osaka, Japan). The relative expression levels of all genes were determined using the 2^–ΔΔ^ *^CT^* (−delta delta CT) method (Schmittgen and Livak, 2008); candidate *Fom-2* regulatory genes were identified through gene expression levels at 0, 24, and 72 h of inoculation in melon, ABA (10 mg/L), MeJA (1.2 mmol/L), and SA (1. mmol/L) induction, respectively.

### Statistical Analysis

All numerical datasets were computed, analyzed for means ± standard deviations, and graphically represented using Microsoft Excel (version 2016). The significant levels were determined by using Student’s *t*-test at *p < 0.05, ^**^p < 0.01, respectively.

## Results

### Phenotypic Observations of Infected Melon Plants

The phenotypic observation of fusarium wilt infection was done in seedlings of both melon varieties (“M4-45” susceptible and “MR-1” resistant) inoculated with *fusarium oxysporum* (f. sp. *melonis* Race 1) at different time points (ACK, A12 h, and A72 h, and BCK, B12 h, and B72 h), respectively (Supplementary Figure 1). There were no initial symptoms appeared in the seedlings of both melon varieties at ACK, A12 h, and BCK, B12 h time points; however, the visual fungal symptoms, necrotic spots and browning in plant roots, and leaf wilting and curling were observed after 72 h of inoculation in M4-45 variety, but no symptoms were observed after B72 h of inoculation in MR-1 variety, respectively.

### Comprehensive Analysis of Transcriptome

A total of 129.99 Gb of clean read data was obtained, and each sample data size comprised up to 6.38 Gb averaged data. The quality base (% ≥ Q30) percentage of data reads was noticed as 91.85% or higher, and GC contents of all samples ranged from 40 to 45% (Supplementary Table 3). The efficiency of comparative reads between each sample and the reference genome was statistically ranged from 79.18 to 91.14%, and 88% of average reads were aligned for each sample, while 86.99% of average reads were compared to the unique locations of the reference genome. The reads compared with multiple sites averaged 1.01%, and the uniform comparison rate between the samples indicated the comparable statistics of samples data. All specific comparisons were matched over the reference genome, and subsequent analysis of the expression level and differential genes was carried out.

**FIGURE 1:**
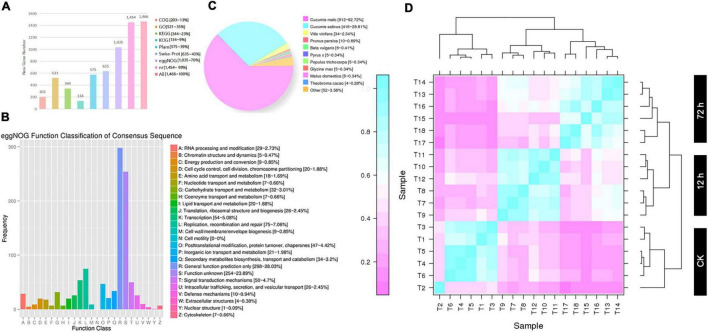
Analysis of new gene annotation and biologically repeated correlation of gene expression among 18 samples. **(A)** Annotation analysis of multiple databases of new genes. **(B)** COG annotation analysis. **(C)** NR annotation analysis. **(D)** The Heatmap of correlation coefficients of 18 samples (T1-T18) of DEGs.

Furthermore, a total of 1,904 new genes were discovered and mainly displayed in GFF files (Supplementary Table 4) and FASTA files (Supplementary Table 5), and these new genes were annotated in each database by the BLAST software (Figure 1A). However, a total of 1,466 genes were functionally annotated; among them, 203 new genes were annotated by the COG library, describing 13% of all annotated genes. In the GO library, a total of 521 new genes were annotated that described 35% of all annotated genes. In the KEGG library, a total of 344 new genes were annotated, which accounted for 23% of all annotated genes. In the KOG library, a total of 134 new genes were annotated, accounting for 9% of all annotated genes. In the Pfam library, a total of 575 new genes were annotated, which accounted for 39% of all annotated genes. In the Swiss-Port library, a total of 635 (43%) new genes were annotated, a total of 1,035 (70%) new genes were annotated in the eggNOG library, and a total of 1,454 (99%) new genes were also annotated in the NR library, respectively (Figures 1B,C).

The gene expression of both materials exhibited significant differences in the disease resistance level at 0 (CK), 12, and 72 h after infection, and expression correlation was divided into three main branches of the evolutionary tree (Figure 1D). Unfortunately, in the CK branch, T2 data showed poor Pearson’s correlation coefficient (<0.6). At the same time period of inoculation in both groups (ACK, A12h, and A72h, and BCK, B12h, and B72h), the overall average of three repeated correlations of the same materials was accurately 0.6, which were located in similar branches of the evolutionary tree and exhibited significant correlations by indicating reliable expression data. However, at 0 (CK), 12, and 72 h, different materials were completely distinguished into different branches of evolutionary trees by indicating that the two materials had different molecular mechanisms of resistance after infection. These differences compel further differential gene analysis to explain the molecular mechanism of resistance material “MR-1.”

### Analysis of Candidate Differentially Expressed Genes

**FIGURE 2:**
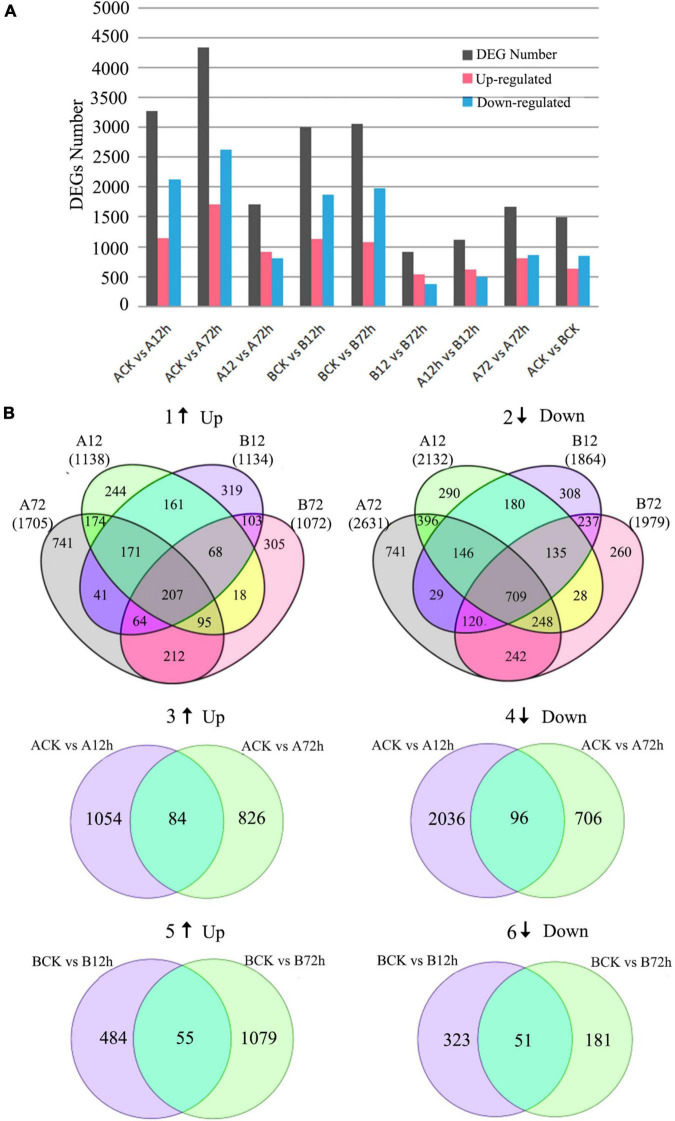
Differentially expressed genes (DEGs) involved in *fusarium oxysporum*-treated melon materials “M4-45 and MR-1” at different time intervals. **(A)** Distribution of identified DEGs. **(B)** Venn diagrams of DEGs; numbers in parentheses represent the total number of upregulated or downregulated genes in each combination, 1, 3, and 5 indicate the upregulated genes expressions, and 2, 4, and 6 indicate downregulated expressions of genes.

For the M4-45 material, 3,270 DEGs (1,138 upregulated and 2,132 downregulated) were identified in response to 12-h post-inoculation (Figure 2A). At 72-h time period, total 4,336 genes (1,705 upregulated and 2,631 downregulated) were identified in control/treatment at FC ≥ 2 and FDR ≤ 0.01. The DEGs analysis between interacting time periods revealed a total of 910 upregulated genes and 802 downregulated genes at A12 h vs. A72 h. For the MR-1 material, a total of 2,998 genes (1,134 upregulated and 1,864 downregulated) were identified in response to12-h post-inoculation. However, at the 72-h time period, a total of 3,051 genes (1,072 upregulated and 1,979 downregulated) were identified in response to control treatment, respectively.

The DEGs analysis between adjacent time periods revealed a total of 539 upregulated genes and 374 downregulated genes at the B12 h vs. the B72 h time period. A total of 442 upregulated genes were identified at 72-h post-inoculation, which might played a positive regulatory mechanism in plant-disease resistance. At the same time period, A12 h vs. B12 h showed that 623 genes were upregulated and 490 genes were downregulated. Furthermore, a total of 807 upregulated genes and 864 downregulated genes were identified for A72 h vs. B72 h. By comparing the above two groups, the difference of expressed genes seemed to be caused by pathogen infection, and changes of upregulated genes expression were also seemed as mediated by disease-resistant genes. It can also be seen that the number of DEGs steadily increased by delaying the *f. oxysporum* inoculation time within the contrasted melon materials (Figure 2A). At the same time, 278 genes were consistently upregulated in the resistant cultivars at initial inoculation of 72-h post-inoculation as compared with susceptible cultivars, and these genes may also play a positive regulatory role in plant disease resistance.

**FIGURE 3:**
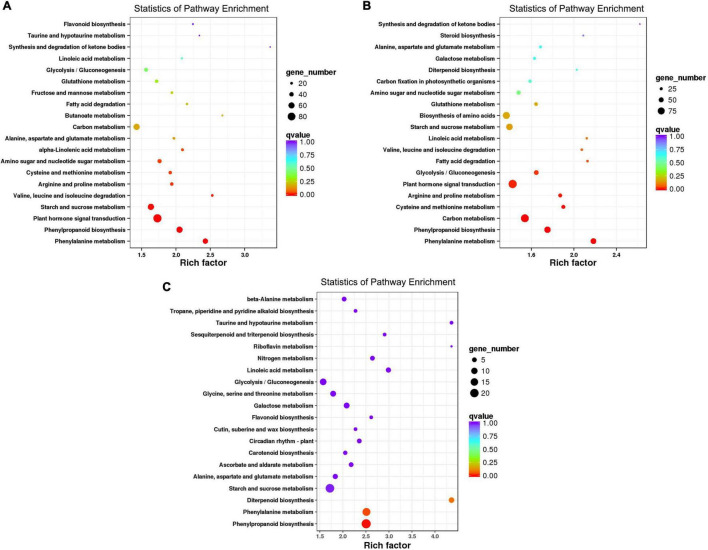
Top 20 enriched pathways analysis. **(A)** DEGs identified in infected the MR-1 cultivar. **(B)** DEGs identified in the infected M4-45 cultivar. **(C)** MR-1 (ACK) vs. M4-45 (BCK).

**FIGURE 4:**
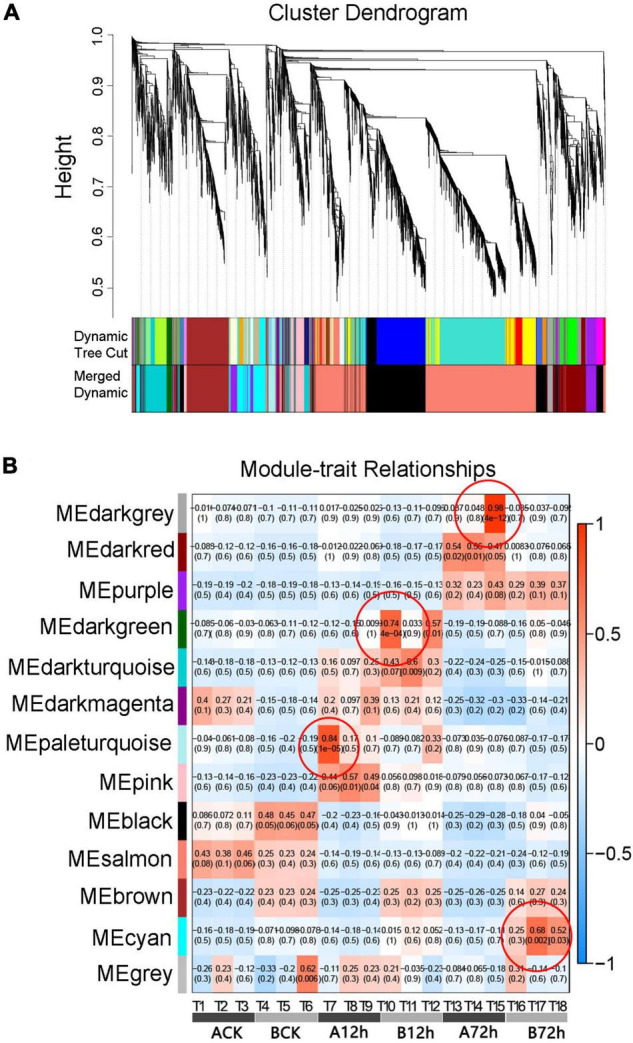
Analysis of WGCNA and module-trait relationships. **(A)** The gene clustering tree and the module-clustering diagram. **(B)** Correlation coefficients between modules and samples.

**FIGURE 5:**
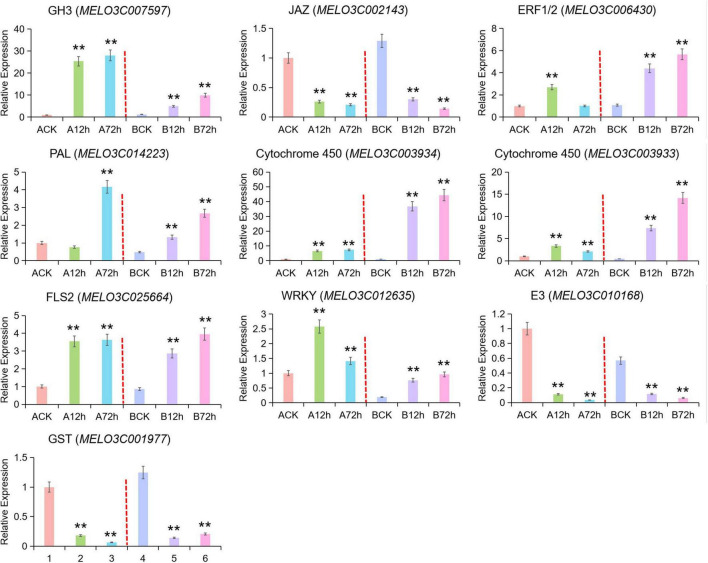
qRT-PCR validation for candidate DEGs across RNA-seq data. Asterick symbol (**) is representing the significant results at **p < 0.01 level, respectively.

**FIGURE 6:**
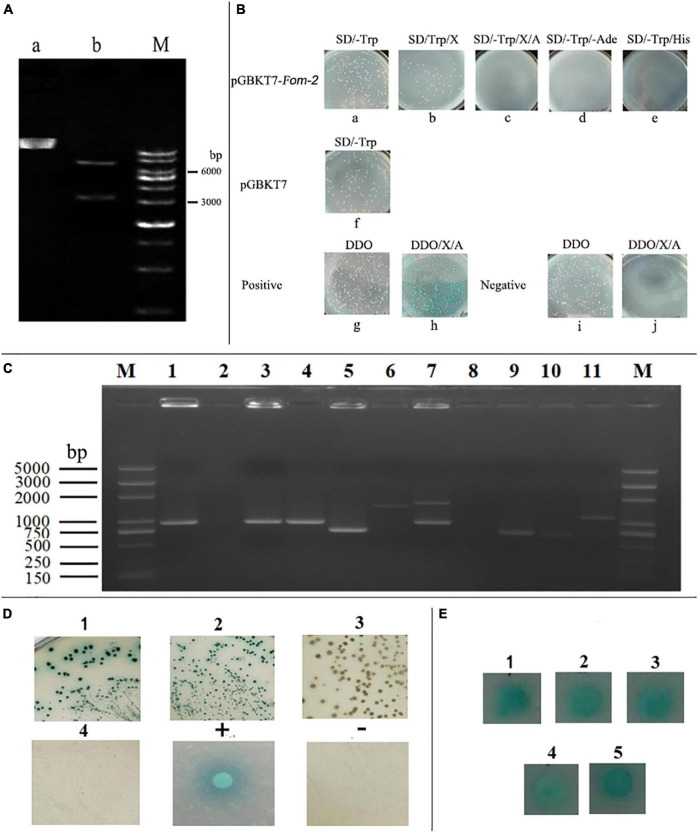
Construction, self-activation, and toxicity identification of *Fom-2* decoy protein. (A) a: pGBKT7-*Fom*-2, b: A double digestion product of pGBKT7-*Fom*-2 and M: 1kb ladder Marker. (B) Detection of auto-activation and toxicity of bait plasmid pGBKT7-*Fom*-2, Positive: Y2HGold (pGBKT7-53) × Y187 (pGADT7-T), Negative: Y2HGold (pGBKT7-Lam) × Y187 (pGADT7-T). (C) Hypothesized positive clones were inserted into yeast plasmid for PCR analysis, M:D2000 plus marker. (D) Interaction was identified by co-transformation of positive clone plasmid, 1: pGBKT7/bait + Candidate prey on DDO/X, 2: pGBKT7/bait + Candidate prey on QDO/X/A, 3: empty pGBKT7 + Candidate prey on DDO/X, 4: empty PGBKT7 + Candidate prey on QDO/X/A, + : positive control on QDO/X/A, −: negative control on QDO/X/A. (E) 1–5 Candidate protein mating verifies on QDO/X.

Furthermore, a Venn diagram for DEGs in response to inoculation at different time periods directly expressed the single and co-expressed DEGs between the samples, respectively (Figures 2B-1,B-2). A total of 607 upregulated genes and 1,170 downregulated genes were co-expressed after 12-h inoculation in two contrasted melon varieties. A total of 578 upregulated genes and 1,319 downregulated genes were co-expressed at 72 h of inoculation. It can be seen that intersection analysis of DEGs expressed at different time points (CK vs. 12 h and 12 h vs. 72 h) of same variety can be used to understand the continuous DEGs by delaying the pathogen infection time, which is beneficial to further analyze the dynamic response of plant-pathogen infection in melon. A Venn diagram analysis similarly showed that there were 180 DEGs (84 upregulated genes and 96 downregulated genes) in the cross set of susceptible cultivars between two groups (Figures 2B-3,B-4). There were a total of 106 DEGs (55 upregulated and 51 downregulated) in the cross set between two groups of disease-resistant cultivars (Figures 2B-5,B-6).

According to the above results, we put these DEGs in | log2Ratio| ≥ 6 as the threshold value of filtering and selection of *f. oxysporum* infection-related expressed genes in melon. Among the total identified DEGs, 50 genes were upregulated and 94 genes were downregulated in M4-45 variety, and a total of 71 genes were upregulated; however, 33 genes were downregulated in MR-1 material, and candidate genes were identified as shown, respectively (Table 1). These genes showed a defense mechanism, secondary metabolism, protein kinases, extension proteins, transcription factors, and unknown proteins. So, we primarily believe that these genes might be related to the melon susceptibility in response to the inoculation. In general, countless genes detection in both cultivars reflects a common response of melon toward the inoculation; however, more number of DEGs seemed to be stimulated in susceptible variety as compared to the resistant, and most of the DEGs were noticed as downregulated in susceptible variety.

**TABLE 1:** Identification of candidate DEGs in analyzed transcriptome data.

Gene ID	Gene description
Cucumis_melo_newGene_36	PREDICTED: lactoylglutathione lyase-like (*Cucumis melo*)
*MELO3C009962*	PREDICTED: polygalacturonase QRT3-like (*Cucumis melo*)
Cucumis_melo_newGene_172	PREDICTED: heat shock 70 kDa protein 16 (*Cucumis melo*)
*MELO3C004428*	PREDICTED: CBL-interacting serine/threonine-protein kinase 6-like (*Cucumis melo*)
*MELO3C008452*	PREDICTED: LOW QUALITY PROTEIN: wall-associated receptor kinase 2-like (*Cucumis melo*)
*MELO3C008453*	PREDICTED: LOW QUALITY PROTEIN: wall-associated receptor kinase 2-like (*Cucumis melo*)
*MELO3C024412*	PREDICTED: putative leucine-rich repeat receptor-like protein kinase At2g19210 (*Cucumis melo*)
*MELO3C003933*	PREDICTED: cytochrome P450 CYP73A100-like (*Cucumis melo*)
*MELO3C019665*	PREDICTED: transcription factor JUNGBRUNNEN 1-like (*Cucumis sativus*)
*MELO3C012635*	PREDICTED: probable WRKY transcription factor 71 isoform X1 (*Cucumis melo*)
*MELO3C006430*	PREDICTED: ethylene-responsive transcription factor 1B (*Cucumis melo*)
*MELO3C009383*	PREDICTED: thaumatin-like protein 1b (*Cucumis melo*)
*MELO3C004385*	PREDICTED: pathogenesis-related protein PR-4-like (*Cucumis melo*)
*MELO3C021623*	PREDICTED: elicitor-responsive protein 1-like isoform X1 (*Cucumis melo*)
*MELO3C005215*	PREDICTED: defensin J1-2-like (*Cucumis melo*)
Cucumis_melo_newGene_5490	defensin-like protein 3 (*Citrullus lanatus*)
*MELO3C013293*	PREDICTED: expansin-like A2 isoform X2 (*Cucumis melo*)
*MELO3C015081*	PREDICTED: expansin-like A1 (*Cucumis melo*)
Cucumis_melo_newGene_849	PREDICTED: glucan endo-1,3-beta-glucosidase, basic isoform- like (*Cucumis melo*)
*MELO3C006070*	PREDICTED: acidic endochitinase-like (*Cucumis melo*)
*MELO3C007750*	PREDICTED: epoxide hydrolase 4-like (*Cucumis melo*)
*MELO3C025684*	PREDICTED: cationic peroxidase 1-like (*Cucumis melo*)
*MELO3C002422*	PREDICTED: uncharacterized protein LOC103485044 (*Cucumis melo*)
*MELO3C026305*	PREDICTED: uncharacterized protein LOC103502424 (*Cucumis melo*)
*MELO3C018228*	hypothetical protein Csa_3G851930 (*Cucumis sativus*)

### Kyoto Encyclopedia of Genes and Genomes Enrichment Analysis of Differentially Expressed Genes

It was found that *f. oxysporum* inoculation caused the significant and different genes expression in disease-resistance-related metabolic pathways involved in both varieties. Among them, the pathways of Phenylalanine metabolism (KO00360), Phenylpropanoid biosynthesis (KO00940), plant hormone signal transduction (KO04075), and plant-pathogen interaction (KO04626) may cause the resistance differentiation between both varieties, having susceptible and resistant mechanisms. After the pathogen infection, the DEGs of MR-1 seemed to be enriched in 117 pathways; out of which, 20 pathways were highly enriched (Figure 3A and Supplementary Table 6). There were a total of 51 DEGs involved in the Phenylalanine metabolic pathway, 61 DEGs were involved in the pathway of Phenylpropanoid biosynthesis, 85 DEGs involved in the plant hormone signal transduction pathway, and 34 DEGs were involved in the plant-pathogen interaction pathway; however, 8 genes were noticed as involved in the flavonoid biosynthesis pathway.

After the inoculation, the DEGs of resistant variety “M4-45” were noticed as highly enriched in 120 pathways; out of which, just 20 pathways were highly significantly enriched (Figure 3B and Supplementary Table 7). A total of 59 DEGs were involved in the L-Phenylalanine metabolic pathway, 67 DEGs were involved in the phenylpropane biosynthesis pathway, 90 DEGs were involved in the signal transduction pathway of plant hormone, and 41 DEGs were involved in the plant-pathogen interaction pathway. However, plant hormone signal transduction and plant-pathogen interaction pathways were not enriched (Figure 3C). Therefore, we categorized that Phenylalanine metabolism (metabolic pathway KO00360), plant hormone signal transduction (KO04075), and plant-pathogen interaction pathway (KO04626) were all enriched by disease-resistance genes, which were mediated by pathogen infection (Supplementary Tables 8–10), respectively.

### Weighted Gene Co-expression Network Analysis of Disease Resistance-Related Differentially Expressed Genes

In order to further analyze the relationship between DEGs related to various transduction pathways in response to inoculation, the WGCNA algorithm was used to conduct the co-expression network analysis for screened DEGs associated with fusarium wilt disease resistance in melon (Figure 4A). It can be seen that all genes are clustered into 13 different modules represented by each different color. The number of genes in each module is allocated according to its expression level, correlation clustering, and the genes assigned to a module have a higher clustering degree. The modules with tissue correlation (>0.65) were defined as tissue-specific modules, and four tissue-specific modules were identified as “dark-gray, dark-green, pale turquoise, and cyan” (Figure 4B). The genes enrichment module analysis exhibited that four modules could be enriched into significant Gene Ontology (GO) pathways, contributing in the biological processes, molecular functions and cell components, and also enriched into regulatory pathways related to disease resistance, indicating that WGCNA depicted biologically significant co-expression modules.

The module-trait relationship analysis is similarly indicated by WGCNA (Table 2), which exhibited that few genes were connected with the disease-resistance mechanism and encode course proteins, transcription factors and protein kinase, benzene propane biosynthesis path, plants-pathogen interaction path, and glutathione S-transferase (GST). The expression of these genes is of great importance because it supports the resistance of melon to fusarium wilt disease, e.g., disease resistance-associated Response protein 206-like, PREDICTED: Elicitor-responsive protein 1-like isoform X1, PREDICTED: Disease-resistance protein RGA2-like. Basically, the expression of pathogen-related protein-like was upregulated in resistant cultivars. The annotated transcription factors include MYC2, WRKY, and MYB, and ethylene responsive transcription factors were noticed to play a very important role in the biological processes of resistance and disease resistance in many plants. In the interaction pathway between plants and pathogen, heat shock proteins (HSP) were continuously upregulated in resistant varieties of Cucumis_melo_newGene_172 and *MELO3C0 25085* after 12-h inoculation. The defensive protein Cucumis_melo _new Gene _5490 and cell wall receptor-associated kinase proteins *MELO3C008446*, *MELO3C008452*, and *MELO3C008453* were continuously upregulated in the resistant variety “MR-1.” The PREDICTED genes related to the phenylpropane biosynthesis pathway are mainly cytochrome P450 cyp73A100-like and PREDICTED: Cytochrome P450 705A5-like (CYTOchrome P450 705A5-like), which are involved in a variety of biochemical reactions and have close relationship with plant-disease resistance. In this study, protein kinase expression was also well-defined after *f. oxysporum* infection in melon, and mainly upregulated in resistant variety after 12 h of inoculation.

**TABLE 2:** Identification of candidate DEGs with a strong correlation module using WGCNA.

*MELO3C016325*	PREDICTED: disease resistance response protein 206-like (*Cucumis melo*)	−−−−−	−−−−−	Up	Up
*MELO3C021623*	PREDICTED: elicitor-responsive protein 1-like isoform X1 (*Cucumis melo*)	−−−−−	Up	Up	Up
*MELO3C023577*	PREDICTED:disease resistance protein RGA2-like (*Cucumis melo*)	−−−−−	−−−−−	Up	−−−−−
*MELO3C023694*	PREDICTED: pathogen-related protein-like (*Cucumis melo*)	Up	−−−−−	Up	Up
*MELO3C006070*	PREDICTED: acidic endochitinase-like (*Cucumis melo*)	Up	−−−−−	Up	Up
*MELO3C021297*	PREDICTED: peroxidase 5-like (*Cucumis melo*)	−−−−−	Up	Up	Up
*MELO3C025684*	PREDICTED: cationic peroxidase 1-like (*Cucumis melo*)	−−−−−	−−−−−	−−−−−	Up
*MELO3C006430*	PREDICTED: ethylene-responsive transcription factor 1B (*Cucumis melo*)	Up	−−−−−	Up	Up
*MELO3C015543*	PREDICTED: ethylene-responsive transcription factor ERF017-like (*Cucumis melo*)	−−−−−	−−−−−	Up	−−−−−
*MELO3C013851*	PREDICTED: transcription factor MYC2-like (*Cucumis melo*)	−−−−−	−−−−−	Up	−−−−−
*MELO3C002228*	PREDICTED: transcription factor MYB1R1-like (*Cucumis sativus*)	Up	Up	Up	Up
*MELO3C012635*	PREDICTED: probable WRKY transcription factor 71 isoform X1 (*Cucumis melo*)	Up	−−−−−	Up	Up
*MELO3C009565*	PREDICTED: calcium-dependent protein kinase 26-like (*Cucumis melo*)	−−−−−	Up	−−−−−	Up
*MELO3C005914*	PREDICTED: CDPK-related kinase 6-like (*Cucumis melo*)	−−−−−	−−−−−	−−−−−	Up
*MELO3C023441*	PREDICTED: probable LRR receptor-like serine/threonine-protein kinase At4g36180 isoform X2 (*Cucumis melo*)	−−−−−	−−−−−	Up	Up
*MELO3C004428*	PREDICTED: CBL-interacting serine/threonine-protein kinase 6-like (*Cucumis melo*)	−−−−−	Up	−−−−−	−−−−−
*MELO3C009224*	PREDICTED: putative receptor protein kinase ZmPK1 (*Cucumis melo*)	−−−−−	−−−−−	−−−−−	Up
*MELO3C024412*	PREDICTED: putative leucine-rich repeat receptor-like protein kinase At2g19210 (*Cucumis melo*)	−−−−−	−−−−−	Up	Up
*MELO3C003933*	PREDICTED: cytochrome P450 CYP73A100-like (*Cucumis melo*)	Up	Up	Up	Up
*MELO3C023530*	PREDICTED: cytochrome P450 705A5-like (*Cucumis melo*)	−−−−−	−−−−−	Up	Up
Cucumis_melo_ newGene_172	PREDICTED: heat shock 70 kDa protein 16 (*Cucumis melo*)	−−−−−	−−−−−	Up	Up
*MELO3C025085*	PREDICTED: 15.7 kDa heat shock protein, peroxisomal (*Cucumis melo*)	−−−−−	−−−−−	Up	Up
Cucumis_melo_ newGene_5490	defensin-like protein 3 (*Citrullus lanatus*)	−−−−−	−−−−−	Up	Up
*MELO3C008446*	PREDICTED: wall-associated receptor kinase 2- like (*Cucumis melo*)	−−−−−	−−−−−	Up	Up
*MELO3C008452*	PREDICTED: LOW QUALITY PROTEIN: wall- associated receptor kinase 2-like (*Cucumis melo*)	−−−−−	−−−−−	Up	Up
*MELO3C008453*	PREDICTED: LOW QUALITY PROTEIN: wall- associated receptor kinase 2-like (*Cucumis melo*)	−−−−−	−−−−−	Up	Up
*MELO3C006355*	PREDICTED: probable glutathione S-transferase (*Cucumis melo*)	−−−−−	−−−−−	Up	Up
*MELO3C015051*	PREDICTED: E3 ubiquitin-protein ligase PUB22 –like (*Cucumis melo*)	Up	Up	−−−−−	Up
*MELO3C008784*	PREDICTED: E3 ubiquitin-protein ligase PUB22 –like (*Cucumis melo*)	Up	Up	Up	−−−−−

In the plants-pathogen interaction pathway, heat shock proteins (HSP) of Cucumis_melo_newGene_172 and *MELO3C025085* were continuously upregulated in resistant varieties at 12 h after infection. The defensive protein Cucumis_melo _new Gene _5490 and cell wall receptor-associated kinase proteins *MELO3C008446*, *MELO3C008452*, and *MELO3C008453* were also continuously upregulated in the resistant varieties. The PREDICTED genes related to the phenylpropane biosynthesis pathway are mainly cytochrome P450 cyp73A100-like and PREDICTED: Cytochrome P450 705A5-like (CYTOchrome P450 705A5-like), which were potentially involved in a variety of biochemical reactions and signified a close relationship with plant-disease resistance. The glutathione S-transferase (*MELO3C006355*) gene was also associated in the module and noticed was continuously upregulated in disease-resistant varieties after pathogen infection, but not showed regulation in the susceptible varieties.

We found that other GST-related genes seemed to be downregulated in the resistant cultivars, although the downregulated trend in susceptible cultivars was stronger than that in resistant cultivars. This GST expression pattern was verified in the fluorescence quantification. It can be known that melon was infected by pathogenic bacteria, its own perception of external stimuli, and signal reception, and signal transmission defense response system was activated, and then the downstream defense response was activated. Moreover, in this study, two E3 ubiquitin ligases were also found to be continuously expressed in high quantities in the susceptible material after pathogen infection. Other types of ubiquitin ligases in differential genes play different roles, including positive regulation of PTI, positive/negative regulation of ETI, positive/negative regulation of immune response, which play an important role in plant disease resistance.

In general, some of the genes analyzed in this part corresponded with some of the genes listed in Table 1, e.g., Cucumis_melo_newGene_172, *MELO3C0044 28*, *MELO3C008452*, *MELO3C008453*, *MELO3C024412*, *MEL O3C003933*, *MELO3C012635*, *MELO3C006430*, *MELO3C016 325*, *MELO3C021623*, Cucumis_melo_newGene_5490, *MELO 3C006070*, and *MELO3C025684*. Some of the genes were only upregulated in resistant variety (*MELO3*Cmelo_newgene_172, *MELO3C008452*, *MELO3C008453*, MELOIS_MELO_newgene_ 5490, *MELO3C016325*, *MELO3C021623*, and *MELO3C024412*), and it can be speculated that these overlapping genes are actively involved in melon’s response to fusarium wilt disease and play a certain regulatory role in the disease resistance process.

### Analysis of Quantitative Polymerase Chain Reaction Expression for Differentially Expressed Genes Validation

In order to verify the accuracy of transcriptome data and to obtain the DEGs, a total of 10 potential genes (GH3, JAZ, ERF1/2, PAL, Cytochrome P450, FLS2, WRKY, E3 Ubiquitin-protein Ligase, GST) related to disease resistance were randomly selected, and qRT-PCR verification was done. The expression pattern results of these genes were basically consistent and showed a similarity index in both analysis of RNA-seq and qRT-PCR (Figure 5), and these results verified the statistical reliability of data. Auxin response-related genes (GH3 and *MELO3C007597*) exhibited as upregulated at 12 h of pathogen infection and gradually increased as the time passed. The upregulated range was stronger in the susceptible cultivars than resistant cultivars. The GH3 gene family belongs to the early auxin responsive genes encoding auxin-binding enzyme that maintains the dynamic balance of auxin content to some extent in plants. The downregulated expression of JAZ in resistant cultivars was the same after the pathogen infection, and the downregulated expression of JAZ and MYC2 genes was slightly higher in resistant cultivars than in susceptible cultivars at 72-h post-infection. The JAZ and MYC2 genes seemed to be downregulated and upregulated after inoculation by indicating the JA receptive mechanism in melon toward the pathogen infection.

The *MELO3C006430* gene encode ethylene response transcription factor (ERF1) in the ethylene signaling pathway of resistant cultivars began to upregulate at 12 h of post-infection and continued the upregulation, while this gene became downregulated at the peak of 12-h post-infection in resistant variety. The *MELO3C014223* gene encoding Phenylalanineammonialyase (*PAL*) was downregulated at 12-h post-infection and then upregulated in susceptible variety, while it was continuously upregulated in resistant varieties with little difference in the expression level. The overall genes expression of cytochrome P450 (*MELO3C003934* and *MELO3C003933*) was continuously upregulated and exhibited as significant in the disease-resistant cultivar, while *MELO3C003933* began to decrease after reaching the peak at 12 h post-infection in susceptible variety. The expression pattern of FLS2 (*MELO3C025664*) in resistant and susceptible varieties was similar by showing an upregulated expression trend. The WRKY (*MELO3C012635*) transcription factor was upregulated at 12 h post-infection in susceptible variety and then downregulated but continuously upregulated in resistant variety. In this part, randomly selected E3 ubiquitin ligase (*MELO3C010168*, u-box type protein PUB22) and GST (*MELO3C001977*) were downregulated in both resistant and susceptible varieties, which also exhibited the downregulated genes enriched at the transcriptional level.

### Analysis of Construction, Self-Activation, and Toxicity Identification of *Fom-2* Decoy Protein

The *Fom-2* target fragment was connected with the decoy vector of pGBKT7 fragment after the double digestion of endonucleases (*Bam*HI and *Eco*RI). The fragment size was detected by bacterial solution, and enzyme-digested products of PCR were verified by AGAR gel-electrophoresis, as shown in Figure 6A. Furthermore, the oligo-synthesized molecular sequencing results showed that the decoy vector was successfully constructed and could be used for self-activation and toxicity verification tests.

After the self-activation culture test, Y2HGold (PGBKT7-*Fom*-2) exhibited the colony growth on SD/-Trp plate (Figure 6B-a). On the SD/-Trp/X plate, the colony growth did not turn blue (Figure 6B-b), indicating the no self-activation of the *MEL1* gene. There was no colony growth on the SD/-Trp/X/AbA plate (Figure 6B-c), the SD/-Trp/-Ade plate (Figure 6B-d), and the SD/-Trp/-His plate (Figure 6B-e), indicating that the AUR1-C gene, the Ade2 gene, and the His3 gene were not self-activated. Meanwhile, positive control showed colony growth on the SD/-LEu/-TRP plate (Figure 6B-g), blue colony growth on the SD/-Leu/-Trp/X/A plate (Figure 6B-h), and white colony growth on the SD/-Leu/-Trp plate (Figure 6B-i), but there was no observation of colony growth in SD/-Leu/-Trp/X/A (Figure 6B-j). So, it can be concluded that PGBKT7-*Fom*-2 has no self-activation in yeast. In terms of colony size, the colony diameter of bait plasmid Y2HGold (pGBKT7-*Fom*-2) grown on the SD/-Trp plate was not significantly different as compared to the empty vector SD/-TRrp plate, and indicated that constructed bait plasmid had no toxic effect on yeast cells (Figure 6B-f). The above analysis further indicated that the decoy plasmid could be further used for screening yeast two-hybrid.

### Screening and Identification of Yeast Two-Hybrid Positive Clones

The mating between yeast strains Y2HGold containing the recombinant bait plasmid PGBKT7-*Fom*-2 and Y187 of a cDNA library was done at 30°C for 20–24 h (30∼50 rpm). At 20 h, a drop of culture was observed at the 40x phase of a contrast microscope, and conjugate was found. The cells were resuspended, and hybridization efficiency was calculated by coating the product on SD/Trp, SD/Leu, and SD/Leu/Trp. The results showed that hybridization efficiency was in an appropriate range and suitable for library screening. The other products were cultured upside down on DDO/X/A plates for 3–5 days at 30°C. A large number of white and blue clones were found, and all the blue clones were inoculated on the DDO/X plate. After the culture, the blue clones still appeared two times and were verified on the QDO/X/A plate.

The 21 blue colonies produced after many rounds of screening were identified using bacterial liquid PCR with the designed primer pGADT7-F/R for plasmid specificity identification. A total of 15 positive clones were amplified with bands, as shown in part 1 of Figure 6C. The bait plasmid, pGBKT7 empty vector, and 15 library plasmids were all co-transformed into Y2HGold yeast cells to reduce false positives. Seven false positives were eliminated when the transporters were coated with DDO/X and QDO/X/A plates. In the end, only 8 library plasmids were co-transformed on the appropriate plates. The presence of blue colonies suggests that the proteins expressed by these 8 library plasmids interact with the decoy proteins (Figure 6D). The positive interaction clones were screened on LB plates containing Amp + resistance after the 8 library plasmids were transformed into DH5 *E. coli*. At the same time, three colonies from each plate were chosen for PCR detection. One of the three identically sized positive clones was chosen at random and sent to Sangong Bioengineering (Shanghai) Co., Ltd., for sequencing.

The gene clone was compared by using the Vecscreen carrier sequences, NCBI,^2^ and the BLASTX database, and can be seen in Table 3. A total of eight gene sequences of the PGBKT7-*Fom*-2 interaction were obtained, including four sequences of the same protein (Ubiquitin-Conjugating enzyme E2) (Figure 6E). The screened proteins interacting with *Fom-2* mainly include transcription factors and important enzymes in the metabolic process. Among them, the screened transcription factors are likely to interact with *Fom-2* and co-regulated the downstream gene expression, including two BZIP transcription factors and two *MYB* transcription factors.

**TABLE 3:** The comparative results of Y2H interaction genes.

No.	Gene ID	Functional description	Chromosome position
1	*MELO3C009678*	MYB transcription factor	chr4: 29092355. 29093746 (+)
2	*MELO3C014597*	MYB transcription factor	chr5: 1070206. 1071795 (+)
3	*MELO3C011839*	BZIP transcription factor	chr10: 4432383. 4435983 (+)
4	*MELO3C019349*	BZIP transcription factor	chr11: 12271096. 12278779 (+)
5	*4XM334FK014*	Ubiquitin-conjugating enzyme E2	chr9: 22698683. 22704152 (+)

### Analysis of *Fom-2* Regulatory Mechanism Against Fusarium Disease

**FIGURE 7:**
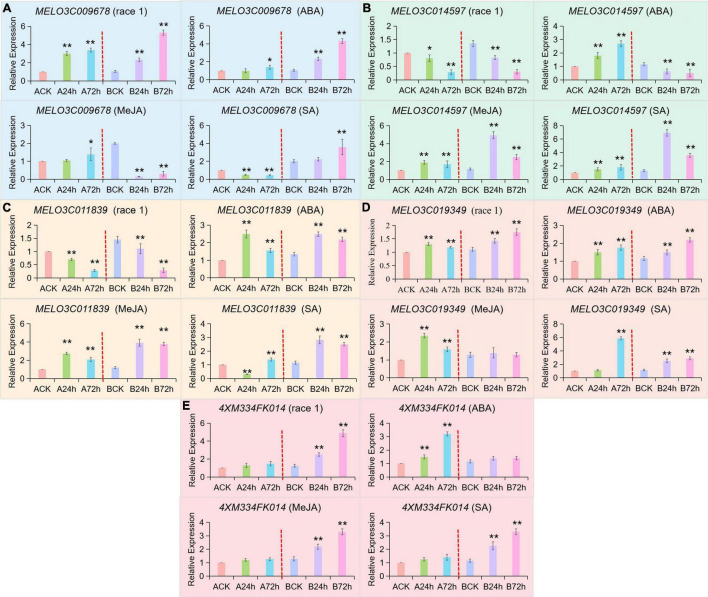
qRT-PCR validation for candidate genes regulating *Fom-2* activity. Asterick symbols (*, **) are representing the significant results at **p < 0.01 and *p < 0.05 levels, respectively. Expression of five candidate genes **(A)** MELO3C009678, **(B)** MELO3C014597, **(C)** MELO3C011839, **(D)** MELO3C019349, **(E)** 4XM334FK014, at different periods of exogenous spraying induction of *f. oxysporum* (Race 1), ABA, MeJA, and SA, respectively.

The fluorescence qRT-PCR analysis of candidate genes expression levels in fusarium wilt (Race 1), and exogenous induction of ABA, MeJA, and SA showed that all the five candidate genes responded to *f*. *oxysporum* stress of ABA, MeJA, and SA induction, as shown in Figure 7. Two MYB transcription factors (*MELO3C009678* and *MELO3C014597*) responded to the fusarium wilt (Race 1) infection signal by showing the opposite regulatory patterns (Figures 7A,B). The *MELO3C009678* gene was significantly upregulated in both of the disease resistant and susceptible materials and shown to be positively regulating the disease-resistance mechanism. However, the *MELO3C014597* gene was significantly downregulated in both materials but reversed the disease-resistance pathway. By exogenous induction analysis of ABA, MeJA, and SA hormones, the *MELO3C009678* gene responded to ABA and SA signals and increased consistently in the resistant materials. The *MELO3C014597* gene responded to MeJA and SA signals, although the expression level of this gene increased first and then decreased in resistant materials, and then an overall increment took place.

Two BZIP transcription factors (*MELO3C011839* and *MELO3C019349*) responded to the infection signal of fusarium wilt (Race 1), but their regulatory patterns were different (Figures 7C,D). The expression of the *MELO3C011839* gene was significantly downregulated in both disease-resistant and disease-susceptible materials but noticed as reversed the resistance pathway. The expression level of *MELO3C019349* gene was stable in both disease-resistant and disease-susceptible materials. By induction analysis of ABA, MeJA, and SA, the expression level of the *MELO3C011839* gene in disease-resistant and disease-susceptible materials was noticed as increased first and then decreased, which was different from that in response to the melon-*fusarium oxysporum* (Race 1) infection signaling. The *MELO3C019349* gene also responded to all hormonal induction, but the expression pattern was different from that of specific melon-*fusarium oxysporum*. It was exhibited that the *4XM334FK014* gene did not respond to the infection signal of fusarium wilt (Race 1) in susceptible cultivar (a *Fom-2*-deficient cultivar) but significantly upregulated the resistant cultivars and positively regulated the resistance pathway (Figure 7E). These results indicated that deletion of *Fom-2* did affect the expression level of *4XM334FK014*, so it was preliminary speculated that *4XM334FK014* was downstream of *Fom-2* and had a certain correlation with the expression pattern in MR-1 in response to hormonal induction of MeJA and SA. In summary, the hormonal induction analysis of ABA, MeJA, and SA exhibited that, among the 5 genes interacting with *Fom-2*, including the *4XM334FK014* gene did not respond to the ABA signal, and *MELO3C019349* did not respond to the MeJA signal, but both responded to the salicylic acid (SA) signal. Hence, it was signified that the SA signal pathway is effectively regulating the *Fom-2* gene activity.

## Discussion

### Transcriptomic Analysis for Candidate Differentially Expressed Genes

In this study, a large amount of transcriptome information was obtained and analyzed after *f. oxysporum* infection in resistant and susceptible melon. The KEGG analysis revealed that a large number of DEGs were identified to be associated with the disease-resistance mechanism in both cultivars, involved in various disease-resistance pathways, e.g., DEGs in MR-1 material were enriched in 117 pathways, and DEGs in M4-45 were enriched in 120 pathways, respectively (Supplementary Tables 6, 7 and Figure 3). These genes specifically include in the plant hormone signal transduction pathway, the Phenylpropanoid synthesis/Phenylalanine metabolism pathway, and plant-pathogen interaction (AMP-triggered immunity system), while the DEGs in the mock control group were not enriched in plant hormone signal transduction and the plant-pathogen interaction pathway. Therefore, we infer that these metabolic pathways are mediated by disease-resistance genes in response to pathogen infection, resulting in the enriched pathway for disease resistance.

The strong correlation module analysis by WGCNA further identified that disease resistance genes have course-related proteins, transcription factors and protein kinase, the benzene propane biosynthesis path, the plants-pathogen interaction path, and glutathione S-transferase (Table 2), and the expression of these genes corresponded with some of the genes listed in Table 1. It can be speculated that these overlapping genes are actively involved in melon’s response to fusarium wilt and play a certain regulatory role in the disease-resistance process. Plant disease resistance is a complex systemic project, different resistance genes have different response genes, and the same gene of different resistance genes response effect is similarly different (Sebastiani et al., 2017). Therefore, it is a great significance to analyze the strong and complex genetic mechanism of candidate genes involved in response towards the disease-resistant of melon.

The plant defense response mechanism is a multiple and complex signal network of intercrossed regulation (Fahad and Bano, 2012; Fahad et al., 2016a,b, c, d; Liu Y. et al., 2016). Plant hormones (GA, ABA, ET, JA, and SA) play key roles in plant resistance pathways; however, ET, JA, and SA have been extensively reported in the specialized plant defense response toward disease resistance (Saud et al., 2014, 2016, 2017, 2020). It has been shown that ET, JA, and SA form an extremely systematic network to regulate the interactions between plants and exogenous stresses (biological or abiotic) by allowing the plants to tolerate the adverse environments (Fahad et al., 2018; Saleem et al., 2020a,b, c). Among them, SA mainly responds to biological stress by mediating plant SAR, while ET and JA respond to biological stress by mediating the induction of systemic resistance (ISR) (Gayatridevi et al., 2012). It was also found that SA specifically inhibits the expression of Auxin-related genes, and one of the mechanisms of SA-mediated defense response is the inhibition of the Auxin signaling pathway that indicates a certain role in the process of plant defense.

Furthermore, ABA is considered to be a negative regulator of plant disease resistance, and its expression level is associated with increased disease sensitivity. It was similarly reported that ABA affects plant responses to biological stress mainly through interactions with other biodefense response pathways (Mauch-Mani and Mauch, 2005; Ul Hassan et al., 2021). ABA is induced by signal or environmental stimulation in plants. It binds to *PYR*/*PYL* receptor protein and then interacts with *PP2C* to release the inhibition of the *SnRK2* positive regulator by *PP2C*, and ABA signaling response is activated (Hu et al., 2012). In this study, the transcripts of *PYR*/*PYL* and *PP2C* were changed after inoculation of fusarium wilt pathogen in melon (Supplementary Table 9), suggesting the relevant results of previously published studies that the ABA signaling pathway was involved in melon response to fusarium wilt pathogen.

JA and ET have been extensively studied in plant-pathogen interactions (Bari and Jones, 2009; Pieterse et al., 2009). JA-mediated signal transduction includes three processes, JA synthesis, JAZ ubiquitination and degradation, and MYC2 activation (Chini et al., 2007). Furthermore, our identified DEGs encoding JAZ and one encoding MYC2 (*MELO3C013851*) (MYC2 was only upregulated in MR-1), suggesting that the pathogen stimulated JA biosynthesis, which also suggested that the JA signaling pathway was involved in melon response to fusarium wilt. At the same time, with the extension of infection time, the differential genes encoding JAZ and MYC2 and their expression levels in MR-1 were higher than those in M4-45, indicating that MR-1 responded more strongly to the JA signaling pathway activated by *fusarium wilt* stress. The transcriptomic study of sunflower in response to verticillium wilt infection revealed that the gene-encoding JAZ was upregulated in the samples treated with susceptible variety P77, but two downregulated genes encoding JAZ were found in the samples treated with resistant variety S18 (Guo, 2017). In the transcriptome study of sugarcane infected by *Ustilago scitaminea*, it was found that both *JAZ* and *MYC2* genes were upregulated, and the expression levels of resistant varieties were higher than susceptible varieties (Su, 2014).

The expression levels of genes in the above studies are different from that of our current study, because genes react differently in different disease resistance pathways in different crops. It was evidently narrated that JAZ and MYC2 genes were upregulated and downregulated in the study of penicillium-infected Hami melon (Shan et al., 2016), which was similar to the results of this study. The MYC2 is an important regulatory factor in the JA signaling pathway for disease and insect-defense response, and the ERF transcription factor in the ethylene signaling pathway antagonistically regulates the expression of downstream disease-defense genes (Ding et al., 2016; Gao et al., 2020). The ethylene response factor ERF located downstream of *EIN3* can specifically bind to promoters of ET resistance-related genes and play a positive role in regulating the expression of resistance genes (Zhang and Dong, 2016), which play a key role in regulating plant immune responses. In this study, the gene encoding ethylene responsive transcription factor (ERF1, *MELO3C006430*) was also noticed as upregulated in both resistant and susceptible cultivars at 12 h of inoculation (Supplementary Table 9). The expression of *MELO3C006430* in resistant cultivars continued to be upregulated with the extension of inoculation time, while the expression of *MELO3C006430* in susceptible cultivars began to decrease, indicating that ET-mediated defense response was involved in the response of melon to fusarium wilt disease. The TGA can induce *PR1* expression in the SA-mediated signal transduction pathway. In this study, the gene encoding (TGA, *MELO3C009647*) was upregulated in the resistant varieties after inoculation, and its expression level decreased gradually with the extension of time. At 72 h of inoculation, *MELO3C004385* (which regulates the disease course-related protein *PR1*) was upregulated in the resistant varieties. This expression pattern is similar to the expression of genes in the study on penicillium infestans in cantaloupe.

The calcium-calmodulin signaling pathway has been proved to be an important component in plant resistance response by a large number of studies (Pandey et al., 2002; Olmos et al., 2003; Fahad et al., 2015a,b). In this experiment, genes related to this pathway were activated by fusarium wilt of melon; for example, calcium-binding EF hand family protein (*MELO3C010876*), calcineurin B-like protein (*MELO3C006022*, *MELO3C008492*, and *MELO3C003339*) (Supplementary Table 10). It was reported that the similar results of the disease resistance-associated signal are responsive (Sun et al., 2019) when plants are stimulated by light, environmental stress, pathogen infection, and plant hormones, and then the concentration of cytoplasmic Ca^2+^ changes, and the stimulated calcium signal is similarly transmitted. CDPK and CML, Ca^2+^ receptors that sense changes in intracellular Ca^2+^ concentration and conduct stress signals, were also differentially expressed in this study, and they all share the same EF hand structure for binding Ca^2+^.

Leucine-rich repeat *LRR* is a class of highly representative pathogen recognition receptors in plants. The *LRR* domain plays an important role in the response process related to plant-disease resistance. It cannot only participate in protein-protein interaction by binding ligands but also similarly promote the signal transduction in defense response (Xie et al., 2019). In this study, most of the genes encoding *LRR* protein were downregulated, and the expression levels of these downregulated genes in the resistant material (MR-1) were lower as compared to the susceptible material (M4-45). The relative gene expression in susceptible cultivar was steadily increased with prolongation of inoculation time, and most of them were similarly downregulated. A total of five genes encoding receptor kinases were identified during the response of melon toward fusarium wilt; two receptor kinases encoding leucine-rich repeat units, *MELO3C024412* and *MELO3C023441*, and three genes encoding cell wall-related receptor kinases, *MELO3C008446*, *MELO3C008452*, and *MELO3C008453* appeared as upregulated in resistant variety (MR-1) (Supplementary Table 10).

### Molecular Mechanism of *Fom-2* Regulatory Gene

The Y2H screening technique was carried out for constructing the melon yeast library infected by pGBKT7-*Fom*-2 bait plasmid and *F. oxysporum* inoculation. Due to the certain false-positive difference in Y2H, the positive clones obtained by screening must be further verified by methods such as a reverse hybridization experiment and immunoprecipitation. In this experiment, we chose the backcross verification method of one-to-one hybridization between the decoy plasmid strains and the strains containing AD plasmids, respectively. Finally, 5 candidate genes were obtained, and we need to conduct the in-depth studies combined with experiments verifying protein interactions, such as pull-down (Table 2). Through bioinformatics analysis of candidate genes, it was found that the proteins encoded by these 5 genes were hydrophilic proteins, mainly involving transcription factors and important enzymes in the metabolic process. The genes “*MELO3C009678* and *MELO3C014597*” belong to the *MYB* transcription factor family, *MELO3C011839* belongs to the BZIP transcription factor family, *MELO3C019349* is an unknown protein but identified as a new protein, and *4XM334FK014* is a ubiquitin ligase E2 type.

Plant resistance against pathogens is affected by transcriptional regulation, which is one of the main techniques to regulate the expression of plant defense response genes, specifically involving in the role of transcription factors (Fahad et al., 2017). When plants are infected by pathogens, transcription factors in plants can be activated by phosphorylation or binding with other regulatory proteins (MYB and BZIP transcription factors) through signal transmission. Transcription factors can regulate the expression of target genes by binding specific cis-acting elements in the upstream promoter region of defense-related genes (Al-Zahrani et al., 2022). Transcription factors are likely to interact with *Fom-2* and co-regulate the downstream gene expression, and these transcription factors are similarly involved in many signaling pathways in plant growth and development by contributing the hormone signaling, pathogen defense, and environmental stress.

The earlier studies have shown that *AtMYB44* plays an important role in biological and abiotic stress in *Arabidopsis thaliana*, regulating ABA-related stomatal closure and drought and salt and osmotic stress responses (Jung et al., 2008). It has also been demonstrated that *AtMYB44* is involved in the *ET* signaling pathway by binding to the *EIN2* promoter region in Arabidopsis thaliana to participate in the regulation of relevant insect resistance response (Lü et al., 2013). It was found that *AtMYB44* positively regulates *Arabidopsis thaliana*’s defense response to Pseudomonas syringae (PstDC3000) through an NPR1-dependent SA defense signaling pathway (Zou, 2013). These results suggested that *AtMYB44*, as an important stress response gene, is involved in plant resistance and defense response. In this study, two *MYB* transcription factors, *MELO3C009678* interacted with *Fom-2*, were screened by the yeast two-hybrid technique, which exhibited significantly upregulation in both disease-resistant and disease-susceptible materials, and noticed for positively regulating the disease-resistance pathway responding toward ABA and SA signals. However, the expression of *MELO3C014597* gene was significantly downregulated in both disease-resistant and disease-susceptible materials by reversely regulating the disease-resistance pathway and responding toward MeJA and SA signals (Figures 7A,B). These two transcription factors jointly respond to the SA pathway that might have an antagonistic mode of interaction with the *Fom-2* gene to participate in the defense response.

The BZIP transcription factor is involved in biological stress response to insect pest and pathogen infection, and rice *OsbZIP1* may enhance resistance to *Magnaporthe grisea* through SA, JA, and ABA signal transduction pathways (Meng et al., 2005). TGA is a member of the subfamily of *BZIP* transcription factors, which converts SAR induction signals into cellular responses by regulating the expression of defense reactively related genes, and direct resistance response genes and enzymes (Uknes et al., 1992). In this study, TGA encoding gene “*MELO3C009647*” screened from transcriptome data analysis seemed to be upregulated in the disease-resistant varieties, suggesting that this gene enhanced melon resistance through the signal pathway after fusarium wilt disease infection (Table 2). Two BZIP transcription factors (*MELO3C011839* and *MELO3C019349*) were screened from yeast two-hybrid (Table 3 and Figure 7C), and the *MELO3C011839* gene was significantly downregulated in both disease-resistant and susceptible materials at 72 h, but it regulated the disease-resistance pathway in a reverse mode. The expression level of *MELO3C019349* was stable in both disease-resistant and disease-susceptible materials (Figures 7C,D). These two genes responded to all hormonal induction and were significantly upregulated at 12 h. However, different from the signal expression pattern in the response to *f. oxysporum* infection in melon, it was speculated that the BZIP transcription factor may indirectly affect the resistance response of muskmelon *Fom-2* gene as an upstream regulatory factor.

Ubiquitination is one of the most common protein modification methods in eukaryotes and plays an important role in plant immune response and resistance-related pathways. The E2 gene is a key enzyme in the process of ubiquitination, which has an effect on many proteins in cells, and it exists in many species and is an important enzyme in the metabolic process. Numerous E2 family members have been identified as follows: a total of 14 ubiquitin E3 ligases in yeast, 41 in Arabidopsis, 52 in maize, 75 in tomato, and 39 in rice (Kraft et al., 2005; Michelle et al., 2009; Wang et al., 2014; Jue et al., 2015; Zhang et al., 2015). At present, ubiquitin E3 ligases have been extensively studied during ubiquitination, while E2 ubiquitin-binding enzymes (UBCs) have been relatively rarely studied. Some studies have shown that E2 UBCs play an important role in regulating the plant growth and development, such as participating in plant response to environmental stress. It was revealed that overexpression of mung bean “Vr UBC1” enhances the osmotic stress tolerance of *Arabidopsis thaliana* (Eunsook et al., 2013). The ubiquitin-binding enzyme E2 gene “Bc UBCE2” was cloned from Chinese cabbage and found that “*Bc UBCE2*” plays an important role in copper stress response (Zhao et al., 2014). The soybean ubiquitin-binding enzyme gene “*Gm UBC2*” was overexpressed in *Arabidopsis thaliana* that significantly enhanced the tolerance to drought and high salt (Zhou et al., 2010). The other ubiquitin E2 protein-binding enzyme (*Cm UBC*) gene was cloned in melon and studied its functional characteristics and expression pattern (Baloglu and Patir, 2014). The results showed that the ubiquitin E2 protein-binding enzyme gene plays an important regulatory role in response to physiological water loss stress (Zhou et al., 2010). However, the response of the ubiquitin E2-binding enzyme UBC gene in melon has not been reported at a deep molecular level.

In this study, it was also found that the *4XM334FK014* (E2) gene in the susceptible material (*Fom-2*-deficient variety “M4-45”) did not respond to the infection signal of melon *fusariam oxysporum* (Race 1), and was significantly upregulated in the resistant variety, positively regulating the resistance pathway (Figure 7E). These results indicated that the deletion of *Fom-2* did affect the expression level of *4XM334FK014*, so it was preliminarily speculated that *4XM334FK014* had a certain correlation with *Fom-2*. Through the hormonal induction analysis of ABA, MeJA, and SA hormones, the *MELO3C014597* gene responded to ABA and SA signals, and the expression pattern of this gene was the same as that of the specific melon infection signal caused by *f. oxysporum*. Hence, we speculated that E2 is mainly involved in the process of the number and activity of transcriptional regulatory proteins. There might be more numbers of protein degradation and abnormal proteins that would be involved in response to melon-*fusarium oxysporum*-induced stress, responsible for regulating the plant cell metabolism, growth, and development process; however, the role of E2 ligase still needs to be focused in the next step of the study.

In crux, we believe that our molecular findings of putative genes would be helpful for in-depth genetic understanding of disease resistance and the susceptible mechanism in melon plants.

## Data Availability Statement

The data presented in this study are deposited in the NCBI repository, accession number: PRJNA842515. The submitted data can be checked at: https://www.ncbi.nlm.nih.gov/search/all/?term=PRJNA842515.

## Author Contributions

TY, JL, and XML conducted the entire experiment, data curation, and formal analysis. SA assisted in the formal analysis, scientific writing, reviewing, and editing of the manuscript. XYL and MZ participated in the molecular experiments. YZ assisted in the theoretical guidance. FL provided the experimental resources and useful practical guidance. HL and XW supervised the research project, reviewed, and edited the manuscript. All authors contributed to the article and approved the submitted version.

## Conflict of Interest

The authors declare that the research was conducted in the absence of any commercial or financial relationships that could be construed as a potential conflict of interest.

## Publisher’s Note

All claims expressed in this article are solely those of the authors and do not necessarily represent those of their affiliated organizations, or those of the publisher, the editors and the reviewers. Any product that may be evaluated in this article, or claim that may be made by its manufacturer, is not guaranteed or endorsed by the publisher.

## Footnotes

1
http://bioinfogp.cnb.csic.es/tools/venny/

2
http://blast.ncbi.nlm.nih.gov/Blast.cgi
