# Supplementary material for: Transcriptomic Analysis of Fusarium oxysporum Stress-Induced Pathosystem and Screening of Fom-2 Interaction Factors in Contrasted Melon Plants
Source: Front Plant Sci. 2022 Jul 22;13:961586. doi: 10.3389/fpls.2022.961586 (PMC9354789; doi:10.3389/fpls.2022.961586)
Supplement: Supplementary file 1 [file Data_Sheet_1.ZIP › Supplementary material/SUPPLEMENTARY FIGURE S1/SUPPLEMENTARY FIGURE S1.docx]

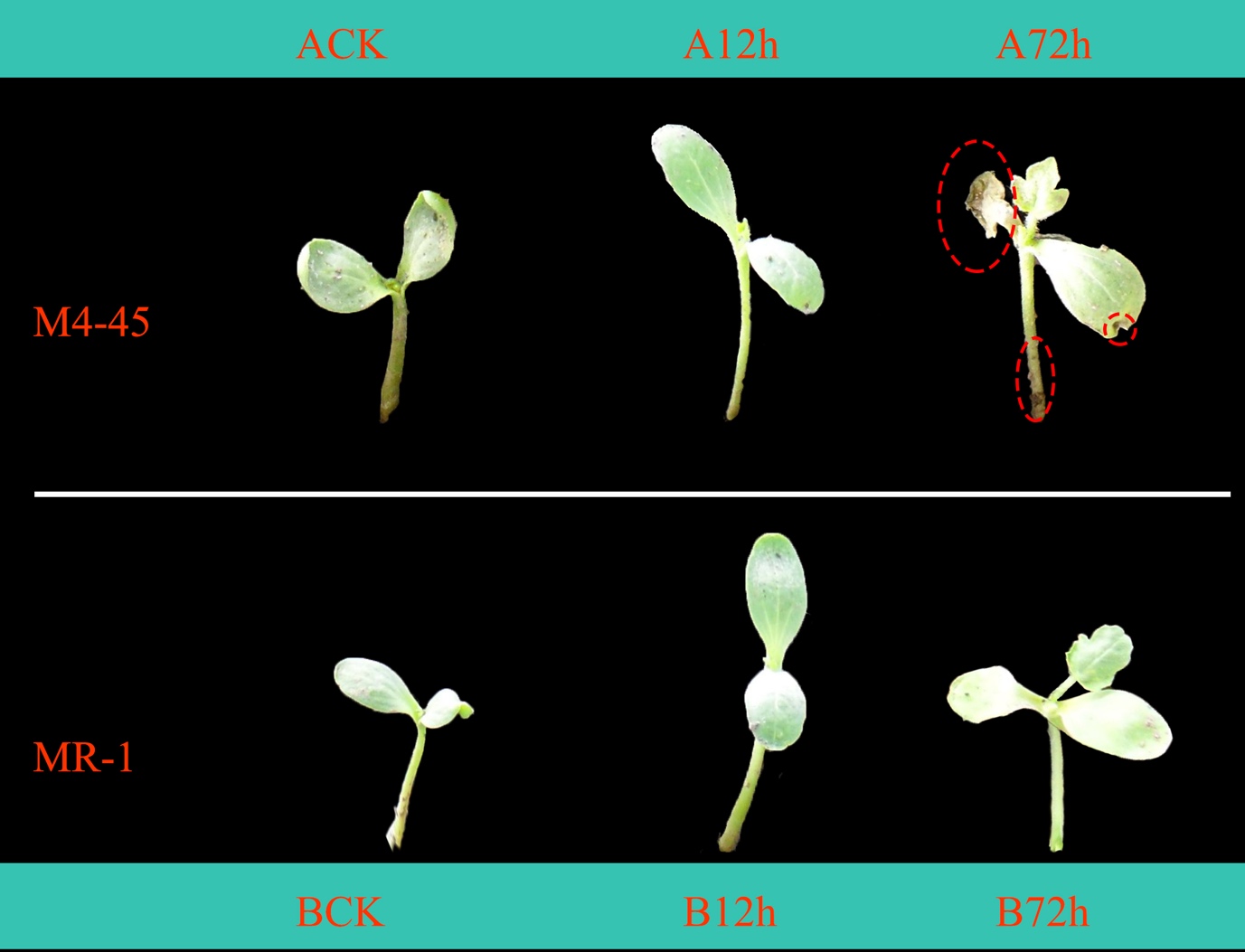


**SUPPLEMENTARY FIGURE S1 |** The visual observation of contrasted melon varieties (M4-45 “susceptible” and MR-1 “Resistant”) at 0 (CK), 12 and 72 hours (h) after *fusarium oxysporum* inoculation. Red circles are indicating the initial typical symptoms of fusarium wilt infection at seedling stage of M4-45 variety.
